# Implementing standardised community-based service package to improve TB outcomes in six countries

**DOI:** 10.5588/pha.25.0021

**Published:** 2025-09-03

**Authors:** M. Volik, L. Tonkonoh, Y. Kalancha, P. Valieva, N. Heydarova, D. Zhurkin, N. Shumskaia, O. Bobokhojaev, S. Naimov, O. Klymenko, S. Hasanova, O. Rucsineanu

**Affiliations:** ^1^ Stichting Tuberculosis Europe Coalition, Utrecht, The Netherlands;; ^2^ Stop TB Partnership (Hosted by the UN Office for Project Services), Geneva, Switzerland;; ^3^ TB Europe Coalition, Nizhyn, Ukraine;; ^4^ TB Europe Coalition, Utrecht, The Netherlands;; ^5^ Saglamliga Khidmat, Baku, Azerbaijan;; ^6^ Republican Research and Practical Centre for Pulmonology and Tuberculosis, Minsk, Belarus;; ^7^ Public Foundation “Den Sooluk Nuru”, Bishkek, Kyrgyzstan;; ^8^ Ministry of Health and Social Protection of the Population of the Republic of Tajikistan, Dushanbe, Tajikistan;; ^9^ Stop TB Partnership, Dushanbe, Tajikistan;; ^10^ CO “TBPeopleUkraine”, Kyiv, Ukraine;; ^11^ WHO Regional Office for Europe, Copenhagen, Denmark;; ^12^ ANB de TB din RM “SMIT”, Bălţi, Moldova.

**Keywords:** tuberculosis, community engagement, Azerbaijan, the Republic of Belarus (Belarus), the Kyrgyz Republic (Kyrgyzstan), the Republic of Moldova (Moldova), Tajikistan, Ukraine

## Abstract

**BACKGROUND:**

Although the need for community-based support services as part of TB care is reaffirmed in various strategies, there are no data on the implementation progress of the recommended standardised package of community-based support services to improve TB outcomes developed by a consortium of partners in 2021.

**METHODS:**

The study describes country adaptation and initial planned implementation of the community-based packages in six countries of Eastern Europe and Central Asia – Azerbaijan; the Republic of Belarus (Belarus); the Kyrgyz Republic (Kyrgyzstan); the Republic of Moldova (Moldova); Tajikistan; and Ukraine – using programme review and qualitative data.

**RESULTS:**

An analysis of the package adaptation and initial implementation is presented from the perspective of the country implementers with a focus on country-specific approaches and lessons learned. The analysis framework is focused on the following specific areas: 1) adaptation practices; 2) ensuring quality and supervision of the services; and 3) securing funding. Commonalities and differences in each of these areas are analysed.

**CONCLUSION:**

In all countries, standardised community-based service packages were adapted and gradually introduced to support clinical TB care. Proper costing and monitoring of the services delivered at the community levels and integrating the budgeted packages into national TB programmes are recommended to ensure sustainability.

The WHO European region has made a significant progress towards the 2025 End TB Strategy milestones.^1,2^ Nevertheless, the high-priority countries in the region still bear a large share of the TB burden, especially of drug-resistant TB, with the highest proportions of multidrug-resistant TB (MDR-TB) found in Eastern Europe and Central Asia (EECA).^2,3^ Acknowledging community-based support services as an integral part of comprehensive people-centred TB prevention and care^1,4^ and the need to scale up the support provided by civil society organisations (CSOs),^5–8^ the standardised package of community-based support services to improve TB outcomes (SPCBSS-TB) was developed to help countries incorporate community-based services into the National TB Programmes (NTPs).^9,10^ The SPCBSS-TB includes a group of 12 non-medical services aiming to address the comprehensive needs of communities and individuals in timely seeking and accepting care and to compliment clinical TB care.

Since its development in 2021, no assessments have been conducted to document country experiences and current progress in the adaptation and implementation of the SPCBSS-TB in the region. This article aims to assess adaptation and planned implementation practices of the community-based packages in six countries of the EECA region to further inform programmatic improvements of the support services in other similar settings.

## METHODS

The study was designed as a mix of qualitative assessments in a variety of settings chosen to obtain a representation of adaptation practices across the EECA region and presents the results of: 1) a programme review, 2) qualitative interview data, and 3) analysis of data collected through community-led monitoring (CLM). The study design and tools were discussed, tested, and agreed within the research team, consisting of national coordinators in each country led by an international expert.

A desk review of the national TB strategic plans, policies related to state social contracting systems, available country-specific reports and documentation related to external funding (current and/or expected), as well as research papers and grey literature was performed, using a template with standard questions. A total of 81 document sources were taken into the desk review – it included national TB strategic plans, government laws and regulations, executive orders, national reports, and Global Fund grant implementation reports.

Semi-structured interviews were conducted with informants identified through a convenience sample, among representatives of local CSOs, national policy makers, contracting agencies, including NTPs, and international development partners, using a specific questionnaire between July and August 2024 with a total of 33 respondents (Table 1). The overall analysis focused on country approaches in adapting the national packages of services; services included in the national packages, criteria for provision and geographic accessibility; quality; tools used for monitoring and evaluation (M&E); training programs for the implementers of the national packages of services; and available financing mechanisms. The CLM data available in the countries were extracted from the CLM open-source platforms: OneImpact for Azerbaijan, Tajikistan, and Ukraine and I Like VST for Moldova. The data collected at the country level were triangulated by the national-level coordinators, who compiled a templated country-specific report and contributed to the consolidation of a joint report. Additionally, the data were analysed across the countries by two members of the team (MV and OR). These data were compared and are presented below under three thematic areas: 1) adaptation of the community package; 2) ensuring quality and supervision; and 3) funding.

**TABLE 1.** Number of respondents per country and their affiliations.

Type of affiliated organisation	Countries						Total
	Azerbaijan	Belarus	Kyrgyzstan	Moldova	Tajikistan	Ukraine	
Government	2	1	1	2	2	2	10
Non-governmental, local	3	3	7	4	2	1	20
International			1			1	2
Non-affiliated/independent	1						1
Other							0
Total responses	6	4	9	6	4	4	33

The need for ethics approval was waived for this study, but written consent was obtained from all key informants.

## RESULTS

The adaptation practices of the community package described in this article were implemented by the non-governmental organisations (NGOs) in coordination with the corresponding NTPs and development partners in the countries.

### Adaptation of the community package

All six countries followed similar approaches in adapting the national packages of services based on country consultation, resource mapping, and joint decision-making. Every country held consultations with key stakeholders to adapt the SPCBSS-TB. This required establishing an expert coalition and included dialogues with representatives of state institutions, primarily ministries of health and NTPs, as well as representatives of civil society and communities. For example, Kyrgyzstan held several rounds of the national dialogue to discuss the service package and ensure its adoption at the country level.

The countries conducted resource mapping to identify existing services, followed by a gap assessment of what would be needed for the components of the national service package. For example, in Moldova, it included the comparison of the existing list and scope of NGO-provided services against the recommended SPCBSS-TB.

The discussion on practical implementation of the services at the community level led to country-specific adaptation of the package by selecting and including those services, which would be potentially implemented at the country level. This was seen as transformation of a minimum set of services initially supported through external grants into a comprehensive national package of standardised services to be provided by the community and civil society. Four countries formally adopted the community package, while in Azerbaijan and Moldova the packages were developed but not yet formalised. Only in Tajikistan, the approved package includes all 12 recommended services; Kyrgyzstan provides 11 services; in three countries – Azerbaijan, Moldova, and Ukraine – 10 services are included in the national packages; and Belarus has 7 services. Non-clinical management of people with TB infection was the most common service not included in the national packages. The informants referred that ‘this particular service is part of a clinical process’ (Table 2; http://dx.doi.org/10.6084/m9.figshare.29382641).

**TABLE 2.** Services included in recommended SPCBSS-TB vs. national community packages, per country (Y = yes; N = no).

Services included in recommended SPCBSS-TB	Services included in national community packages					
	Azerbaijan	Belarus	Kyrgyzstan	Moldova	Tajikistan	Ukraine
Awareness raising, risk communication, community engagement and mobilisation	Y	Y	Y	Y	Y	Y
Counselling people at risk of TB	Y	Y	Y	Y	Y	Y
Non-clinical management of latent TB	N	N	N	N	Y	N
Support for active case finding	Y	Y	Y	Y	Y	Y
Supervised treatment support	Y	Y	Y	Y	Y	Y
Management of loss to follow-up and prevention of treatment interruption	Y	N	Y	Y	Y	Y
Assessment of individual needs of people	Y	Y	Y	Y	Y	Y
Mental health and psychological counselling and support	Y	Y	Y	Y	Y	Y
TB case management	N	N	Y	Y	Y	Y
Material support	Y	N	Y	Y	Y	Y
Health education and counselling	Y	Y	Y	Y	Y	Y
Social support and/or rehabilitation after completion of treatment	Y	N	Y	N	Y	N
Total services included	10	7	11	10	12	10

The analyses show that implementation of the national service packages was often limited geographically. In Moldova, the NTP annually reviewed the priority districts, based on epidemiological situation, and set the targets and territories for the interventions to be covered by NGOs. Therefore, it was necessary to have either an interested NGO in each region or hire new people from those regions to be able to meet the requirements. For example, in Kyrgyzstan, additional TB employees were added to the currently operational NGOs to cover an extra area. Also, due to several projects running in the country, geographic coverage for individual projects was often divided and the list of services varied between projects funded by different donors. Additionally, national packages of services included specific populations for whom services could only be provided. In Kyrgyzstan, the state social contracting system announced in 2023 focused on two groups, lost to follow-up and people on treatment with risk of treatment interruption, while externally funded programmes focused on providing social support solely to people with MDR-TB. Even if support services are recommended as part of the TB package and should be equally available for all, regardless of epidemiologic priorities, strains of TB, these practices revealed challenges in practical implementation of the community packages.

### Quality and supervision

#### Quality assurance

As part of the structured interview, the importance of a central coordinating body and the development of standard practices, as well as national monitoring and evaluation plans, were cited among the key elements of the quality assurance system. In all the countries, at different stages of development of community-based services, quality assurance efforts were initially undertaken within the framework of donor-funded activities. The adaptation of the community packages of services allowed countries to rethink existing programmes for people affected by TB and increasingly focus on the quality of services. Moldova developed and approved standard operation procedures related to the national package of services, including the services related to active TB case finding as well as to improving treatment adherence. As part of these efforts, NTPs in collaboration with community implementers systematically reviewed the target populations, priority settings, and working algorithms, followed by the Ministry of Health’s endorsement.

#### Training programmes

Providing support services at the community level requires staff to have specialised training, skills, knowledge, and competencies. The assessment revealed several approaches used by countries: 1) recommended but not mandatory trainings on various aspects of TB management, community engagement, and service delivery; 2) donors-led and often project-oriented programmes; 3) organisational programmes dependant of the availability of resources; 4) individual trainings opportunities at own expense; and 5) mandatory trainings for (social) workers upon hiring. For example, in Ukraine, the social support employees were mandated to complete a certified online training course, both pre-service and annually in-service. In case of a low score, the social worker must undergo several rounds of training. In Belarus, state social procurement required the community organisation to independently hire only qualified staff. It was found that in all countries assessed, the competencies for the community providers were not sufficiently defined and documented with no systematic approach to training and few cases of capacity building focused on specific services.

#### Monitoring and evaluation

When implementing supportive services, NGOs in countries complied with the existing M&E systems based on: 1) NTP indicators; 2) donor-funded projects indicators; 3) monitoring visits; and 4) internal and external evaluation tools, including CLM and client satisfaction surveys. However, it varied from the absence of a unified mechanism mentioned in Belarus, to the availability of two M&E systems, due to the separation of sources of funding in Azerbaijan. In the case of Belarus, the quality of the individual services was assessed by ensuring that the order and content of their provision corresponded to the algorithms or regulations set for individual services. The government in Azerbaijan additionally required detailed reports, especially for awareness-raising events, which needed to include photo evidence. In the case of Moldova, the NTP partially used the WHO-NGO-related indicators in its work plans, highlighting the importance of their integration into the national planning aimed at improved accountability and sustainability.

### Funding

#### External funding

As a rule, the initial funding of NGO activities to provide services to people with TB in all six countries came from external sources and was provided within the framework of the implementation of donor-supported programmes. The Global Fund remained the main source of external funding in all countries. In some countries, it was also contributed by USAID, the Stop TB Partnership, as well as smaller donor projects, varying from one donor in Moldova to four in Kyrgyzstan (Table 3).

**TABLE 3.** Sources and mechanisms of funding for services included in national community packages, implemented by non-governmental organisations, 2021–2024.

Country	Domestic	External
Azerbaijan	Grants to implement social state contracts distributed by the Agency for State Support for Non-Governmental Organizations of the Republic of Azerbaijan on a competitive basis	1) Global Fund to Fight AIDS, Tuberculosis and Malaria; 2) Stop TB Partnership’s grants
Belarus	Subsidies to implement social state contracts, distributed by local administrations	1) Global Fund to Fight AIDS, Tuberculosis and Malaria grants
Kyrgyzstan	Grants to implement social state contracts, managed by the National TB Center and concluded on a competitive basis	1) Global Fund to Fight AIDS, Tuberculosis and Malaria; 2) United Nations Development Programme; 3) United States Agency for International Development; 4) Stop TB Partnership grants
Moldova	Contracts, concluded, on a competitive basis to implement TB screening services among key population groups, distributed through the Preventive Fund of the National Medical Insurance Company	1) Global Fund to Fight AIDS, Tuberculosis and Malaria grants
Tajikistan	Grants to implement social state contracts distributed by the Ministry of Health and Social Protection of Population of the Republic of Tajikistan on a competitive basis.	1) Global Fund to Fight AIDS, Tuberculosis and Malaria; 2) United States Agency for International Development; 3) Stop TB Partnership grants
Ukraine	Social contracting in the form of well-established mechanism of social services purchasing budgeted and administered by local governments (of any level)	1) Global Fund to Fight AIDS, Tuberculosis and Malaria; 2) United States Agency for International Development grants

#### Domestic funding

In the context of changing funding landscape and preparation for donor withdrawal, all countries mentioned the importance of domestic funding provided at the local community level: from subsidies from the state budget to grants and social contracts, concluded though various procurement mechanisms (Table 3). An important factor noted by the informants was that the funding for the national packages required also updates in the regulatory framework to ensure compliance with the social contracting requirements. Therefore, in recent years, all six countries highlighted the role of state social procurement in various forms (grants, subsidies, or contracts) as well as stable increase in the volume and share of domestic financing in supporting community-based TB services.

#### Costing of services

The SPCBSS-TB includes recommendations for costing of services, allowing countries to use the standardised approach. For example, community-based TB screening was costed and programmed in Moldova. However, none of the countries utilised costing for the entire package of services. In the case of Ukraine, the estimates resulted from the costing exercise conducted in the country were perceived as excessively high and, therefore, not approved at the national level.

## DISCUSSION

The standardised package of community-based support services to improve TB outcomes was developed by TB partners to guide the engagement of civil society actors in service delivery by providing practical tools and by outlining a set of recommended standards for community-based, non-medical services. Here, we examined the experiences in the adaptation of the recommended package at country levels and current progress in six countries of the EECA region, as well as discussed implementation considerations that should be prioritised for further successful implementation of community supportive packages.

All countries had largely discussed the package within inclusive dialogues and adapted practices of community-based support services into national packages ranging from 7 to 12 recommended services per package. The most common service not included in national packages was non-clinical management of people with TB infection. This contrasts with the latest WHO policy guidance,^11,12^ which highlights the role of communities in expanding access to TB preventive treatment from one side and shows existing overall misconception of this role in the provision of this service from the other side. This assessment also found that the planned implementation of the national packages of services was limited to certain regions or specific populations with services often divided and varying between projects funded from different sources. Quality assurance efforts were cited as key but varying across the countries with the majority of the countries reporting several training opportunities but no systematic approach to staff capacity building, calling for the establishment of certified continuous training for personnel who provide services included in national packages, which should become part of the NTP’s human resource development plan. Additionally, the availability of different sources of funding led to different M&E systems to be followed in settings, challenging the overall implementation of the services, at the community level. Furthermore, the recommended M&E indicators for the services provided at the community level, as per the existing regional framework,^10^ are not used, limiting the overall understanding of the impact of community-based interventions. We observed limited or selective use of the costing tool, in opposite of what is recommended by the SPCBSS-TB. This highlighted the critical need of the standardisation and costing of a complete list of services considering the actual need at country levels towards building a sustainable approach in engaging civil society and communities in the provision of supportive TB services, ideally to be integrated in the overall NTP budget.

There are several important limitations to this study. Disaggregation at the subnational levels was not possible due to limited data availability. Scarce quantitative data, including CLM data, and considerable variations in reporting practices provided limited evidence on some of the elements that were assessed, including the coverage, quality of the services, and volumes of funding dedicated to community packages. Future work could seek to include more specific frameworks to evaluate progress in the implementation of the community supportive package as well as the impact of support services on improving the effectiveness of TB care and the expectations of the ultimate consumers of the services.

Despite these limitations, this assessment has navigated the stages of the SPCBSS-TB adaptation and planned implementation in six EECA countries, shedding light on the practical application of the recommended service package. The experiences of the countries show that the adaptation and implementation of the national community-based supportive packages is feasible and needs to be continued with improved training programmes for community implementers, aligned with national commitments to sustaining funding for the community packages implementation and most importantly with robust frameworks that would allow measuring the added value of those services for the quality of life of people.
